# Psychometric analysis of the Psychiatric Diagnostic Screening Questionnaire (PDSQ) and determinants of psychopathology in two outpatient clinics in Navarre (Spain)

**DOI:** 10.23938/ASSN.1043

**Published:** 2023-08-30

**Authors:** Juan I. Arrarás, Manuel J. Cuesta, Victor Peralta, Gustavo J. Gil-Berrozpe, Laura Barrado, Olga Correa, Rebeca Elorza, Lorea González, Irma Garmendia, Lucía Janda, Patricia Macaya, Camino Núñez, Pablo Sabater, Aileen Torrejon

**Affiliations:** 1 Servicio Navarro de Salud-Osasunbidea Rehabilitation Unit Servicio Navarro de Salud - Osasunbidea Pamplona Spain; 2 Servicio Navarro de Salud-Osasunbidea Institute for Health Research of Navarre (IdiSNA) Pamplona Spain; 3 Servicio Navarro de Salud-Osasunbidea Department of Psychiatry Hospital Universitario de Navarra Servicio Navarro de Salud - Osasunbidea Pamplona Spain; 4 Servicio Navarro de Salud-Osasunbidea Mental Health Department Servicio Navarro de Salud - Osasunbidea Pamplona Spain

**Keywords:** PDSQ, Questionnaire, Psychometric analyses, Determinants, Mental disorder, PDSQ, Cuestionario, Estudio psicométrico, Determinantes, Trastorno mental

## Abstract

**Background:**

The self-report Psychiatric Diagnostic Screening Questionnaire PDSQ is designed to screen Axis I psychiatric disorders. We aim to determine its psychometric properties in Spanish outpatients and assess its relationship with two interviews (for psychopathology and for personality disorders) and clinical/demographic variables.

**Methodology:**

We administered the study questionnaire, the Mini International Neuropsychiatric Interview Plus (MINI-Plus), the Standardised Assessment of Personality Abbreviated Scale (SAPAS), and the List of Threatening Experiences Questionnaire (LTE-Q) to 375 patients at two public outpatient centres. Reliability of the study questionnaire was evaluated (Cronbach’s alpha, α) and known-group validity measured by comparing groups based on demographic and clinical variables (binary logistic regression analysis) and MINI-Plus diagnoses (Mann-Whitney U). The diagnostic accuracy of the study questionnaire score was analysed taking the MINI-Plus diagnoses as the gold standard (ROC analysis).

**Results:**

Internal consistency was adequate across all PDSQ scales (α >0.7; mean α=0.85). Known-group comparisons were satisfactory. Female and male patients showed higher prevalence of internalizing and externalizing diagnoses, respectively. Younger age, more life events and limitations, higher SAPAS scores, and lower economic levels were linked to a greater number of PDSQ diagnoses. Inter-group differences were found for all PDSQ scales based on the corresponding MINI-Plus diagnoses. Mean values of sensitivity, AUC, and negative predictive value were 88.7, 0.82, and 96.7, respectively.

**Conclusions:**

When applied to a sample of Spanish outpatients, the PDSQ exhibits satisfactory psychometric properties and adequate relationships with the psychopathology and personality interviews, and clinical and demographic variables. The study questionnaire is suitable for assessing comorbidity and psychopathology dimensions.

## RESUMEN

**Fundamento:**

El PDSQ (*Psychiatric Diagnostic Screening Questionnaire)* es un cuestionario autoadministrado para el cribado de diagnósticos psiquiátricos del eje I. El objetivo es estudiar sus propiedades psicométricas en pacientes ambulatorios españoles y analizar su relación con dos entrevistas (de psicopatología y de trastornos de personalidad), y con variables clínicas y demográficas.

**Material y métodos:**

Se administraron los instrumentos PDSQ, MINI-Plus, SAPAS, y LTE-Q a 375 pacientes en dos centros ambulatorios públicos. Se estudió la fiabilidad del PDSQ (α de Cronbach). La validez de grupos-conocidos se analizó comparando sub-grupos organizados por variables demográficas y clínicas (regresión logística binaria) y por diagnósticos MINI Plus (U de Mann-Whitney). Se estudió el desempeño diagnóstico del *PDSQ* considerando los diagnósticos MINI Plus como *gold standard* (análisis ROC).

**Resultados:.:**

La consistencia interna del *PDSQ* fue adecuada en todas las escalas (α >0,7; media=0,85). Las comparaciones entre grupos-conocidos fueron satisfactorias. Mujeres y hombres mostraron prevalencias mayores de trastornos internalizantes y externalizantes, respectivamente. Una menor edad, más sucesos vitales y limitaciones, puntuaciones mayores en SAPAS y niveles económicos más bajos se relacionaron con mayor número de diagnósticos PDSQ. Los grupos basados en los correspondienes diganósticos MINI-Plus difirieron en todas las escalas del PDSQ. Los valores medios de sensibilidad, AUC y valor predictivo negativo fueron 88,7; 0,82 y 96,7, respectivamente.

**Conclusiones:**

En su aplicación a pacientes españoles ambulatorios, el PDSQ muestra, propiedades psicométricas satisfactorias y relaciones adecuadas con entrevistas de psicopatología y personalidad, así como con variables clínicas y demográficas. El PDSQ es adecuado para evaluar comorbilidad y dimensiones de psicopatología.

## INTRODUCTION

A high percentage of patients attending Mental Health Services have more than one psychiatric diagnosis^1^. Reliable psychiatric diagnosis of comorbidity is key to provide adequate treatment^2^.

Clinicians tend to under-recognize comorbidity in mental health routine settings when unstructured clinical interviews are used in comparison to structured research evaluations^2^^,^^3^.

Semi-structured interviews, such as the Mini-International Neuropsychiatric Interview (MINI)^4^ or the Structured Clinical Interview for DSM (SCID)^5^, are considered *gold standards* for assessing comorbidity, but they may take more time than available in daily clinical work^6^. Screening scales, like the DSM-5 Cross-Cutting Symptom Measure^7^, may offer better comorbidity assessment than unstructured clinical interviews^3^^,^^8^.

The Psychiatric Diagnostic Screening Questionnaire (PDSQ) is a self-report instrument designed to screen for the most common Axis I psychiatric disorders (as outlined in the DSM IV) in outpatient mental health settings. It is based on the most-often recorded and reported diagnoses in community surveys and clinical samples. The developers of the PDSQ aimed to address the under-recognition of comorbidity in daily clinical practice associated with unstructured interviews^8^^-^^11^. It is understood that PDSQ cannot offer the same degrees of reliability and validity as structured interviews when making diagnoses. However, it offers a reasonable estimate of the overall prevalence of commonly encountered conditions^3^.

The PDSQ is intended to be administered and scored before the patient’s initial visit with the clinician in order to improve the efficiency of the diagnostic evaluation by guiding clinicians towards areas that require more assessment^8^^,^^11^^,^^12^.

The PDSQ is considered a good screening and diagnostic aid due to its capacity to identify comorbidities. It shows good psychometric properties, described by the authors of the scale^2^^,^^8^^,^^10^^,^^11^^,^^13^^,^^14^ and in different countries^15^^,^^16^. The PDSQ has been translated into Spanish using a forward-backward translation process and validated for its use in Spanish alcohol-dependent patients^6^.

The PDSQ shows a positive relationship with other diagnostic instruments such as the SCID^8^^,^^16^ and the Personality Assessment Inventory (PAI)^9^, and has been employed to study the validity of other instruments assessing depression and anxiety^17^.

The PDSQ has been administered in: 1) comorbidity studies of patients reporting substance use^15^, heavy youth drinking^18^, or borderline personality disorder^19^; 2) studies of pregnant and postpartum women^20^ and patients suffering from pain^21^; and 3) studies of patients with specific mental health disorders such as panic disorder^22^, psychotic symptoms^23^, subclinical generalized anxiety disorder^24^, post-traumatic stress^25^ or suicidal ideation^26^. It has also been used to create a network structure of self-reported psychopathological dimensions in common mental disorders^27^.

Although empirical support for the PDSQ has grown, a broader empirical grounding is needed to comprehensively establish its validity^9^. The authors of the PDSQ^8^, for example, recommend replicating the psychometric study in public community health centres because it was originally developed in a private adult practice, where there may have been a lower prevalence of other diagnoses such as chronic and persistent mental illness or patients with lower economic status. Perkey et al.^9^ suggest that further research should focus on convergent and divergent validity with other established psychopathology instruments introducing criteria such as life space data (e.g., education) and personality measures.

In this study, we aim to determine the psychometric properties of the PDSQ when applied to a sample of Spanish outpatients from two public centres. We also explored the relationship between the PDSQ and two established interviews for psychopathology (MINI-Plus) and for personality disorders (SAPAS), as well as the relationship between the PDSQ and clinical and demographic variables

## METHODOLOGY

### Participants

Participants were a consecutive sample of patients who attended one of two outpatient centres of the Mental Health Network of Navarre between June 2012 and March 2016. The inclusion criteria were: patients older than 16 years, referred from the General Practitioner consultation, and undertaking their first consultation at either of the two participating mental health centres. Patients with organic mental disorders or whose cognitive levels prevented them from completing the questionnaires were excluded.

### Data-collection procedures and instruments

All patients who met the inclusion criteria were interviewed, given oral and written information about the study, and invited to participate. Patients who agreed to participate and signed the informed consent were asked to complete three self-reported scales (without any medical professional being present) before their initial diagnostic evaluation consultation at the outpatient centre.

1) The *Psychiatric Diagnostic Screening Questionnaire* (PDSQ)^8^ analyses current and recent symptoms (previous two weeks and six months before evaluation), as well as whether the patient has ever experienced or witnessed a traumatic event. The PDSQ is a 125-item instrument consisting of 13 scales that allows obtaining information from six areas (Appendix I). The resulting global score (from 0 to 100) provides information on the severity of the overall psychopathology. Participants responded to each item using a yes/no format (yes=1 / no=0). Each scale is scored as a dichotomous variable indicating the presence or absence of the diagnosis based on the cut-off points established by the authors or as a continuous variable (that can be converted to a 0-100 scale), where higher scores indicate a greater number of symptoms on that scale^8^; thus, scales may be considered psychopathology dimensions.

2) The *Standardised Assessment of Personality, Abbreviated Scale* (SAPAS)^28^ is is an eight-item interview used to screen the presence of personality disorders, rather than the specific type of these. Each question is to be answered with a yes or no (yes =1 / no =0). The resulting score ranges from 0 to 8; a higher score indicates a greater likelihood of having a personality disorder. A score ≥ 3 indicates the presence of a personality disorder according to the SCID^30^.

3) The *List of Threatening Experiences Questionnaire* (LTE-Q)^29^, or Brugha’s scale, assesses the presence of major stressful life events with considerable long-term contextual threat in the preceding six months. This instrument has been validated in Spain^31^. It comprises 12 items with yes/no answers (yes =1 / no =0). Total scores range from 0 to 12 (higher scores indicate a greater number of life events). The LTE-Q also assesses the degree of limitation or negative consequences (none =0, low =1, middle =2, and high =3) caused by each event, providing a global value (0 to 36); higher scores indicate a greater number of events and degree of limitations.

During this first consultation, the treating psychiatrists (who have previously been trained in administering the PDSQ) also administered the *Mini-International Neuropsychiatric Interview-Plus* (MINI-Plus 5.0.0)^4^, a structured and standardized diagnostic interview used to determine the presence of the 23 most common Axis I psychiatric disorders (DSM-IV-TR and ICD-10). The MINI-Plus is the MINI version created for research. Once the interview concluded, the interviewers indicated the presence or absence of any disorder. For this study, we administered the Spanish version of the questionnaire^32^.

The PDSQ has been previously assessed in a sample of Spanish alcoholic patients. However, we wanted to know whether the wording of the items was adequate for other diagnostic groups. Thus, the first 40 patients were invited to complete a short debriefing questionnaire on the time they had taken to complete the PDSQ instrument, and asked if they had found any of the items confusing or upsetting.

The following socio-demographic data were obtained from study participants:

- gender: female, male;
- age (years);
- household composition: living alone, living with family, shared home, residence;
- educational attainment: less than compulsory, compulsory, post-compulsory below university, university;
- place of birth: Spain, other;
- economic status: low, medium-low, medium, high.

The study was approved by the Drug Research Ethics Committee of Navarre and conducted in accordance with the ethical principles outlined in the Declaration of Helsinki.

### Statistical analysis

Clinical and demographic characteristics and questionnaire scores were presented as frequency and percentage or mean and standard deviation (SD).

The *internal consistency reliability* of the scales was measured by Cronbach’s alpha (α); the internal consistency was considered as adequate if α >0.70^33^.

Assessment of questionnaire validity was performed by known-group comparison to discriminate between subgroups of patients. First, we used binary logistic regression analysis using PDSQ dichotomous scores (which indicate the presence/absence of a diagnosis) as dependent variables.

Explanatory (independent) variables were age, gender, economic level (two groups: 1= medium-low, medium and high; 2= low), stressful life events and limitations caused by these events (LTE-Q scores), and personality (SAPAS scores).

A higher age was expected to be related to less anxiety and drug abuse/dependence, and more major depressive disorders^1^^,^^34^^,^^35^^,^^36^. A higher number of internalizing diagnoses (major depressive disorder, anxiety, bulimia, hypochondriasis, and somatoform disorders) were expected in female patients and a higher number of externalizing diagnoses (alcohol and drug abuse/dependence) in male patients^34^^,^^35^^,^^37^. A higher rate in a PDSQ diagnosis was expected to associate to a lower economical level^38^, a greater number of life events and limitations caused by these events^39^^,^^40^, and a greater likelihood of a diagnosis of personality disorder in general^41^^,^^42^.

Known-group comparison was also performed by comparing the PDSQ scores in each scale of the questionnaire (considered continuous variables ranging from 0 to 100) for groups that presented the corresponding diagnoses in the MINI-Plus interview. The Mann-Whitney U test was performed because PDSQ scores did not follow a normal distribution (Kolmogorov-Smirnov, p <0.001 for all PDSQ areas); effect size was calculated based on Cohen’s D.

PDSQ diagnoses were compared to those obtained by the MINI-Plus interview (gold standard). Diagnostic accuracy of the PDSQ was analysed by means of sensitivity, specificity, positive and negative predictive values (PPV, NPV), and area under the curve (AUC) from ROC analysis. The authors of the PDSQ suggest a sensitivity ≥ 90% for using the scale in clinical practice^8^.

## RESULTS

Of the 394 patients invited to participate in the study, 375 (95.2%) completed the PDSQ. The reasons for not completing the questionnaire were patient refusal (n=10), not having glasses (n=4), difficulty in understanding Spanish (n=2), lack of time (n=2), and intellectual disability (n=1). All patients who completed the PDSQ, answered over 95% of the items. When an answer was missing, we selected the most frequent answer (yes/no) in their corresponding scale of the PDSQ.

**Table 1:** Sociodemographic and clinical characteristics of the patients of the study

Characteristics	n	%
*Gender*		
Female	224	59.7
Male	151	40.3
Age* (range 16-86)	40.2	13.9
*Household composition*		
Living alone	42	11.2
Living with family	301	80.3
Shared home	29	7.7
Residence	3	0.8
Educational attainment		
Less than compulsory	40	10.7
Compulsory	144	38.4
Post-compulsory	135	36.0
University level	56	14.9
Country of birth		
Spain	309	82.4
Other	66	17.6
*Economic status*		
Low	154	41.1
Medium-Low	112	29.9
Medium	71	18.9
High	38	10.1
*LTE-Q*		
Stressful life events* (range: 0-9)	1.3	1.6
Degree of limitation* (range: 0-22)	3.1	4.3
SAPAS* (range 0-7)	3.9	1.6
*Diagnosis*^*1*^		
Major depressive disorder	125	33.3
Post-traumatic stress disorder	33	8.8
Bulimia	5	1.3
Obsessive-compulsive disorder	21	5.6
Panic disorder	68	18.1
Psychosis	22	6.1
Agoraphobia	35	9.3
Social phobia	50	13.3
Alcohol abuse	47	12.5
Drug abuse	38	10.1
Generalized anxiety disorder	70	18.7
Somatization disorder	38	10.1
Hypochondriasis	24	6.4
Other diagnoses (total)^2^	154	41.1
Anxiety-depression mixed disorder^2^	22	6.1
Dysthimia^2^	23	6.1
Suicidal risk^2^	33	8.8
Adaptative disorder^2^	45	12.0

*: mean and standard deviation; 1: diagnoses based on MINI-Plus interview, patients may have more than one diagnosis; 2: DSM IV diagnoses (based on MINI Plus interview) not included in the PDSQ scale; the most frequent diagnoses in this group are presented.

Overall, it took patients under 20 minutes to complete the PDSQ and no item was considered upsetting or confusing. Three patients who had a diagnosis different from psychosis were surprised to be asked about psychotic symptoms because they considered those specific questions had nothing to do with their situation. However, this was not sufficient reason to change the wording of those items, since the patients did not report being upset by them.

Mean age of study patients was 40.2 years (range: 16-86 years), 80.3% of them lived with their family, and 41.1% reported a low economic level. Mean number of stressful life events was 1.3, with a mean SAPAS score of 3.9. Results of the MINI-Plus interview revealed various diagnoses, the most common being major depressive and anxiety disorders (Table 1).

### Descriptive statistics of the PDSQ questionnaire

Median number of comorbidities (PDSQ diagnoses per patient) was 4.0: major depressive disorder, the various anxiety disorders (posttraumatic stress, panic, and generalized anxiety), obsessive-compulsive disorder, and somatization disorder (in decreasing frequency). A PDSQ diagnosis of psychosis was found in 28.5 % of the study sample (Table 2).

**Table 2:** Frequencies in the *Psychiatric Diagnostic Screening Questionnaire* (PDSQ) scales, reliability and comorbidity

	N (%)	Mean (SD)	Floor (%)	Ceiling (%)	Cronbach’s α (95%CI)
Comorbidities (range 0-13)		4.8 (3.1)			
*PDSQ scales*					
Mayor depressive disorder	200 (53.3)	41.9 (25.8)	7.2	0.2	0.90 (0.89-0.92)
Post-traumatic stress disorder	168 (44.8)	30.9 (30.9)	32.5	0.7	0.89 (0.87-0.91)
Bulimia	41 (10.9)	17.0 (26.8)	56.8	0.8	0.86 (0.83-0.88)
Obsessive compulsive disorder	179 (47.7)	14.9 (21.5)	52.3	1.1	0.73 (0.69-0.78)
Panic disorder	132 (35.2)	34.3 (35.7)	36.3	9.3	0.85 (0.83-0.88)
Psychosis	107 (28.5)	10.6 (20.7)	71.2	0.3	0.77 (0.73-0.80)
Agoraphobia	139 (37.1)	24.9 (27.4)	38.1	1.3	0.85 (0.83-0.87)
Social phobia	188 (50.1)	29.6 (27.9)	26.7	0.8	0.85 (0.83-0.88)
Alcohol abuse or dependence	88 (23.5)	11.5 (25.7)	76.8	4.0	0.90 (0.88-0.91)
Drug abuse or dependence	86 (22.9)	10.5 (24.3)	77.9	2.9	0.90 (0.88-0.91)
Generalized anxiety disorder	184 (49.1)	56.1 (35.9)	14.1	16.5	0.87 (0.85-0.89)
Somatization disorder	175 (46.7)	31.4 (31.1)	36.0	5.3	0.76 (0.72-0.80)
Hypochondriasis	119 (31.7)	17.1 (29.7)	67.7	4.5	0.86 (0.83-0.88)
PDSQ total		29.9 (17.3)	0.3	0	

Comorbidities: number of PDSQ diagnoses per patient; mean: mean score on the scale (0-100); SD: standard deviation; floor %: percentage of patients who reported the lowest score; ceiling %: percentage of patients who reported the highest score; CI: confidence interval.

Total mean score for the PDSQ was 29.9. The highest mean scores (corrected to a scale of 0 to 100) were for major depressive disorder, panic disorder, and generalized anxiety disorder (Table 2).

The percentage of respondents at floor (patients with the lowest scores) was low for major depressive and generalized anxiety disorders, and for total PDSQ score, and high for psychosis and alcohol and drug abuse/dependence. Percentage of responders at ceiling (patients with the highest scores) was low for most scales. The whole range of scores was presented for all individual scales. Total PDSQ score ranged between 0 and 80.8.

*Internal consistency reliability* was rather good (α > 0.85) for most PDSQ scales except psychosis, somatization, and obsessive compulsive disorder (α > 0.7); mean α= 0.85 (Table 2).

*Known-group comparison*: significant relationships were found among the variables. A higher number of generalized anxiety disorder, social phobia, and drug abuse/dependence diagnosis was seen in younger patients. Gender differences were found for eight scales; higher prevalence of major depressive, anxiety disorders, bulimia, and somatization disorders was observed in female patients, while male patients showed higher prevalence of alcohol and drug abuse/dependence. Lower economic status was associated to higher number of diagnoses in obsessive compulsive disorder and drug abuse or dependence. Higher number of life events and limitations was linked to higher number of diagnoses in nine PDSQ scales. Higher SAPAS score was related to higher number of diagnoses in nine scales (Table 3).

**Table 3:** Association between independent factors and presence of each *Psychiatric Diagnostic Screening Questionnaire* (PDSQ) scale by logistic regression analysis

	Age	Gender (female)	Economic status (low level)*	TLE-Q		Personality (SAPAS)
				Life events	Degree of limitation	
Mayor depressive disorder	0.99	1.53	1.29	1.38	1.11	1.39
	(0.97-1.01)	(1.01-2.32)	(0.86-1.96)	(1.19-1.61)	(1.05-1.18)	(1.21-1.59)
Post-traumatic stress disorder	0.99	1.29	1.25	1.37	1.13	1.34
	(0.98-1.01)	(0.85-1.95)	(0.83-1.89)	(1.18-1.58)	(1.07-1.20)	(1.16-1.57)
Bulimia	0.98	6.42	1.27	1.25	1.10	1.59
	(0.95-1.01)	(2.63-15.65)	(0.66-2.44)	(1.05-1.49)	(1.04-1.17)	(1.27-2.01)
Obsessive compulsive disorder	0.99	1.72	1.59	1.25	1.10	1.36
	(0.98-1.01)	(1.14-2.61)	(1.05-2.41)	(1.09-1.43)	(1.01-1.17)	(1.19-1.56)
Panic disorder	1.01	1.93	1.20	1.24	1.09	1.32
	(0.98-1.02)	(1.24-3.03)	(0.78-1.85)	(1.09-1.42)	(1.04 -1.15)	(1.15-1.52)
Psychosis	0.99	1.18	1.24	1.30	1.10	1.34
	(0.98-1.02)	(0.75-1.89)	(0.79-1.96)	(1.23-1.49)	(1.05-1.16)	(1.15-1.57)
Agoraphobia	0.99	1.15	1.45	1.09	1.04	1.13
	(0.98-1.01)	(0.75-1.78)	(0.95-2.22)	(0.96-1.25)	(0.98-1.07)	(0.99-1.29)
Social phobia	0.98	1.47	1.04	1.11	1.05	1.36
	(0.96-0.99)	(0.97-2.23)	(0.69-1.56)	(0.97-1.26)	(0.99-1.09)	(1.19-1.56)
Alcohol abuse or dependence	0.99	0.28	0.82	1.12	1.04	1.12
	(0.98 - 1.01)	(0.17-0.47)	(0.50-1.34)	(0.97-1.30)	(0.99-1.09)	(0.97-1.31)
Drug abuse or dependence	0.96	0.34	2.18	1.20	1.08	1.10
	(0.95-0.99)	(0.21-0.56)	(1.34-3.55)	(1.10-1.46)	(1.02-1.13)	(0.95-1.28)
Generalized anxiety disorder	0.98	1.97	1.16	1.27	1.09	1.36
	(0.96-0.99)	(1.29-3.90)	(0.78-1.76)	(1.10-1.45)	(1.03-1.15)	(1.19-1.57)
Somatization disorder	0.98	2.01	1.10	1.16	1.07	1.26
	(0.98 - 1.01)	(1.32-3.07)	(0.73-1.66)	(1.02-1.32)	(1.02-1.12)	(1.10-1.44)
Hypochondriasis	1.01	0.95	1.51	1.02	1.01	1.10
	(0.99-1.02)	(0.61-1.47)	(0.97-2.34)	(0.89-1.17)	(0.96-1.06)	(0.96-1.326)

OR: *odds ratio*; CI: confidence interval; values in bold: a significant relationship was found between an independent factor and a PDSQ area. TLE-Q: *List of Threatening Events Questionnaire*; SAPAS: *Standardised Assessment of Personality, Abbreviated Scale*; *: economic level was grouped on two levels: medium-low, medium and high levels (n= 221) and low level (n= 154).

For patients diagnosed with a disorder on the MINI-Plus interview, the values on the corresponding PDSQ scales were significantly higher (with effect sizes ranging from 0.59 to 2.18) (Table 4).

**Table 4:** Inter-group differences in *Psychiatric Diagnostic Screening Questionnaire* (PDSQ) scores based on the presence of a diagnosis using the MINI-Plus

PDSQ scale	MINI-Plus			Effect size*
	N (%)	Yes	No	
		Mean (SD)	Mean (SD)	
Major depressive disorder	125 (33.3)	58.8 (20.9)	33.5 (23.8)	1.12
Post-traumatic stress disorder	33 (8.8)	66.7 (17.4)	27.5 (29.8)	1.6
Bulimia	5 (1.3)	88.0 (13.1)	16.1 (25.6)	3.48
Obsessive compulsive disorder	21 (5.6	33.3 (18.1)	13.8 (21.2)	0.97
Panic disorder	68 (18.1)	58.8 (34.6)	28.8 (33.6)	0.87
Psychosis	23 (6.1)	35.5 (24.4)	8.9 (19.5)	1.17
Agoraphobia	35 (9.39	57.7 (25.9)	22.1 (25.9)	1.16
Social phobia	50 (13.3)	56.1 (23.9)	25.5 (26.4)	1.23
Alcohol abuse or dependence	45 (12.9)	49.3 (38.2)	6.1 (17.8)	1.44
Drug abuse or dependence	38 (10.1)	59.2 (33.2)	5.1 (15.4)	2.18
Generalized anxiety disorder	70 (18.1)	75.8 (27.5)	51.6 (36.1)	0.59
Somatization disorder	38 (10.1)	55.2 (19.6)	28.7 (30.9)	0.97
Hypochondriasis	24 (6.4)	60.0 (33.6)	14.2 (27.1)	1.47

PDSQ: Psychiatric Diagnostic Screening Questionnaire; MINI-Plus: Mini-International Neuropsychiatric Interview-Plus; N: frequency of patients diagnosed with the MINI-Plus interview in the same category as the PDSQ scale; Yes/No: PDSQ scores of patients with (or without) the corresponding disorder when using the MINI-Plus; SD: standard deviation; *: Cohen’s D effect size; all p-values (Mann-Whitney U tests) were < 0.001.

Mean sensitivity of the thirteen PDSQ scales was 88.7%, six of which had a sensitivity >0.90. All scales had a negative predictive value ≥0.90. The average specificity score was 69, and mean AUC 0.82 (Table 5).

**Table 5:** Diagnostic accuracy of the *Psychiatric Diagnostic Screening Questionnaire* (PDSQ) and its scales based on the diagnoses using the MINI-Plus as gold standard

Scale	PDSQ score				
	Sensitivity (%)	Specificity (%)	PPV (%)	NPV (%)	AUC (95%CI)
Major depressive disorder	87	63	54	90	0.78 (0.74-0.83)
Post-traumatic stress disorder	100	60	19	100	0.84 (0.79-0.89)
Bulimia	100	90	12	100	0.97 (0.95-1.0)
Obsessive compulsive disorder	100	55	12	100	0.80 (0.74-0.86)
Panic disorder	65	72	33	90	0.74 (0.67-0.80)
Psychosis	87	75	19	98	0.84 (0.77-0.92)
Agoraphobia	86	68	21	98	0.79 (0.73-0.87)
Social phobia	94	57	25	98	0.80 (0.74-0.86)
Alcohol abuse or dependence	79	84	42	96	0.84 (0.77-0.91)
Drug abuse or dependence	95	85	42	99	0.92 (0.87-0.98)
Generalized anxiety disorder	79	58	30	92	0.68 (0.62-0.75)
Somatization disorder	89	58	19	98	0.77 (0.71-0.82)
Hypochondriasis	92	72	18	99	0.86 (0.78-0.93)
Mean	88.7	69	26.7	96.7	0.82

MINI-Plus: *Mini-International Neuropsychiatric Interview-Plus*; PPV: positive predictive value; NPV: negative predictive value; AUC: area under curved obtained from the ROC analysis; CI: confidence interval; mean**:** average for all the thirteen PDSQ scales.

## DISCUSSION

Here, we present the results of a psychometric study of the PDSQ applied to a sample of Spanish outpatients from two public centres. We have also analysed the relationship between the PDSQ and the MINI-Plus and SAPAS interviews, and clinical and demographic variables.

The high level of completion rate, the low number of missing items, the lack of items considered upsetting or confusing, and the heterogeneity in education levels of the study sample indicates that the questionnaire was well accepted and item presentation adequate. Our results confirm that the PDSQ can be incorporated in outpatient daily clinical practice without causing disruption^9^. The percentage of unanswered items was similar to that reported elsewhere^20^. The time required to complete the questionnaire supports its developers’ aim to help clinicians attain accurate comorbidity diagnoses in a time-efficient manner^9^.

The high percentage of patients living with their families may be an issue specific to our country^43^^,^^44^.

The high frequency of comorbidities observed in our study is in line with those recorded in prevalence studies for our country^45^^,^^46^ and supports the PDSQ as a suitable screening tool^9^. These comorbidity levels are probably favoured by the current classification systems (DSM5 and ICD), where symptoms can be common to several disorders. The PDSQ is considered a suitable tool for studying the relationships between diagnoses and the dimensions that may underlie psychopathology^9^, such as those proposed by Watson et al. for emotional disorders^47^.

In this study, the variable comorbidity is used merely to describe the study sample (a population with a high degree of comorbidity). Our sample therefore represents people with this feature and no modelling or testing has been affected by its use.

The highest prevalence and mean scores for mayor depression and anxiety disorders found with the PDSQ are in line with studies on the prevalence of these disorders among the Spanish general population^34^^,^^35^. A rather high prevalence of somatization disorder and lower prevalence of psychosis (with a low mean score) were expected, since study patients were attending outpatient centres^48^. The differences in some PDSQ scales (such as obsessive compulsive disorder) in relation to the frequency of cases and mean scores may be related to the low cut-off criterion for these scales.

The higher prevalence of possible diagnoses with the PDSQ compared to the MINI-Plus interview may be explained by the role of the PDSQ as a screening tool for guiding clinicians towards areas that need to be evaluated in detail during consultation^2^^,^^8^^,^^10^.

The differences in frequency of respondents at floor for the PDSQ scales - low for major depressive and generalized anxiety disorders and high for psychosis, alcohol and drug abuse or dependence - may be due to the fact that the symptoms of the first group of scales can be present in several diagnoses, whereas the second group of symptoms are more diagnosis-specific. These floor levels, combined with the wide range of scores in the PDSQ scales and total score, as well as the low ceiling effects, indicate that the questionnaire may be a good assessment tool when the scales are considered as psychopathology dimensions with different severity levels (from normality to sub-threshold and full diagnosis)^8^.

Reliability analysis results were satisfactory and in line with those found by the developers of the PDSQ and in psychometric studies conducted in Spain and Romania. Although the alpha coefficients of somatization, psychosis, and obsessive compulsive disorder scales were low in some studies, in ours these three scales reached the 0.7 cut-off value for alpha.

Known-group validity analyses are supported by the results from the comparisons of patient subgroups. Studies of the Spanish general population^34^^,^^35^ and other contexts^49^ have reported that prevalence of social phobia decreases as patients grow older. Prevalence of generalized anxiety disorder also decreases with age^37^.

Unlike the results of the present study, a relationship between age and depression has been observed in two studies performed with the Spanish general population^,^^34^^,^^35^. The relationship between increasing age and lower prevalence of drug consumption found in our study is in line with a study conducted by the Spanish Ministry of Heath^36^. No relationship between age and alcohol abuse/dependence was found among the Spanish general population^1^.

The observed gender differences are in line with those found among the Spanish general population, where female patients have a higher risk of internalizing diagnoses (anxiety and mood disorders) and male patients a higher risk of externalizing diagnoses (alcohol abuse/dependence)^1^^,^^34^^,^^35^.

The relationship between economic level and frequency of diagnosis is in line with the results of the Spanish National Health Survey^50^ - which reported a higher frequency of diagnosis among lower social classes - and with a study of the Spanish general population, which communicated earning gaps between individuals with and without mental disorders^38^.

In our study, there is a clear relationship between life events and the limitations caused by these events and the presence of a diagnosis. A recent meta-analysis indicates correlations of 0.33 and 0.35 between stressful life events and internalizing and externalizing psychopathology, respectively^39^. Moreover, negative life events have been associated to agoraphobia, panic, generalized anxiety, major depressive and generalized anxiety disorders, as well as alcohol and drug abuse/dependence^40^.

The relationship between a higher SAPAS score and a higher frequency of diagnosis is in line with some studies; Huang et al.^41^^,^^42^ found that 51.2% of the patients with a personality disorder were diagnosed with at least one other mental problem.

As in the Romanian validation study^16^, known-groups validity is also supported by the differences in all inter-group PDSQ scales based on the corresponding MINI-Plus diagnosis.

The sensitivity, specificity, negative predictive values, AUC coefficients, and mean values in our study are satisfactory and in line with those found by the authors of the questionnaire, though in our case the bulimia and psychosis scales have higher sensitivity values (difference ≥ 0.13) and the panic scale lower sensitivity values (difference ≥ 0.12)^8^. High sensitivity and moderate specificity mean values were also reported in the Spanish study with alcoholic patients^6^ and the validation study for Romania^16^.

Most PDSQ scales meet the 0.90 sensitivity coefficient recommended by the developers of the questionnaire or were close to it. This threshold, as well as a high negative predictive value and a rather high specificity value, is recommended so that all cases are detected (to avoid false negatives). Although these criteria may favour the presence of false positives, these are considered less of a problem for a screening questionnaire since the main problem is the time clinicians need to determine a lack of a diagnosis^2^. Moreover, the authors of the PDSQ suggest that the presence of false positives may partly be related to the presence of subthreshold forms of the disorders^8^.

There are several strengths to this study: its sample size, the fact that the patients were from public outpatient mental health centres, the analysis with established psychopathology (MINI-Plus) and personality disorder (SAPAS) interviews, and clinical and demographic data. Contrarily, this study could have benefited from a longitudinal design that measured psychopathology (frequencies and dimensions) before and after an intervention, so that responsiveness to changes could be analysed. Further studies should assess psychometric and comorbidity in patients with psychosis to determine the utility of the PDSQ in this population.

In conclusion, the PDSQ has satisfactory psychometric properties when applied to a sample of Spanish outpatients from two public centres in Navarre. It also shows satisfactory relationships with established psychopathology and personality interviews, and clinical and demographic variables. The PDSQ is a suitable tool for assessing a diagnosis and determining the dimension of the psychopathology.

## APPENDIX I

**Table:** The six areas of the *Psychiatric Diagnostic Screening Questionnaire* (PDSQ), containing 13 scales and 125 items

Mood disorders	
Major depressive disorder	21 items
Anxiety disorders	
Post-traumatic stress disorder (PTSD)	15 items
Obsessive compulsive disorder	7 items
Panic disorder	8 items
Agoraphobia	11 items
Social phobia	15 items
Generalized anxiety disorder	10 items
Substance use disorders	
Alcohol abuse/dependence	6 items
Drug abuse/dependence	6 items
Eating disorders (bulimia/binge-eating disorders)	
Bulimia	10 items
Somatoform disorders	
Somatization disorder	5 items
Hypochondriasis	5 items
Psychosis screening	
Psychosis	6 items
